# Arabidopsis NAC Domain Proteins, VND1 to VND5, Are Transcriptional Regulators of Secondary Wall Biosynthesis in Vessels

**DOI:** 10.1371/journal.pone.0105726

**Published:** 2014-08-22

**Authors:** Jianli Zhou, Ruiqin Zhong, Zheng-Hua Ye

**Affiliations:** Department of Plant Biology, University of Georgia, Athens, Georgia, United States of America; University of Massachusetts Amherst, United States of America

## Abstract

One of the most prominent features of xylem conducting cells is the deposition of secondary walls. In Arabidopsis, secondary wall biosynthesis in the xylem conducting cells, vessels, has been shown to be regulated by two *VASCULAR-RELATED NAC-DOMAIN* (*VND*) genes, *VND6* and *VND7*. In this report, we have investigated the roles of five additional Arabidopsis *VND* genes, *VND1* to *VND5*, in regulating secondary wall biosynthesis in vessels. The *VND1* to *VND5* genes were shown to be specifically expressed in vessels but not in interfascicular fibers in stems. The expression of *VND4* and *VND5* was also seen specifically in vessels in the secondary xylem of the root-hypocotyl region. When overexpressed, VND1 to VND5 were able to activate the expression of secondary wall-associated transcription factors and genes involved in secondary wall biosynthesis and programmed cell death. As a result, many normally parenchymatous cells in leaves and stems acquired thickened secondary walls in the VND1 to VND5 overexpressors. In contrast, dominant repression of VND3 function resulted in reduced secondary wall thickening in vessels and a collapsed vessel phenotype. In addition, VND1 to VND5 were shown to be capable of rescuing the secondary wall defects in the fibers of the *snd1 nst1* double mutant when expressed under the *SND1* promoter. Furthermore, transactivation analysis revealed that VND1 to VND5 could activate expression of the GUS reporter gene driven by the secondary wall NAC binding element (SNBE). Together, these results demonstrate that VND1 to VND5 possess functions similar to that of the SND1 secondary wall NAC and are transcriptional regulators of secondary wall biosynthesis in vessels.

## Introduction

Vascular plants have two specialized tissues, xylem and phloem, for the transport of water and food throughout the plant body. The conducting cells in the xylem are tracheary elements that are further grouped into two types, tracheids and vessels. Tracheids are the first type to evolve and are found in seedless vascular plants and gymnosperms, whereas vessels were proposed to have evolved from tracheids and are found in angiosperms [1]. Xylem formation involves a series of complex developmental events, including differentiation of procambial/cambial cells into xylem mother cells, cell elongation, secondary wall thickening and programmed cell death. Molecular and genetic analyses have uncovered a number of important genes controlling the various developmental events of xylem formation [2]. One of the best-studied events of xylem formation is secondary wall thickening in which a cascade of transcription factors has been revealed to be involved.

A transcriptional network involving NAC and MYB transcription factors has been demonstrated to regulate secondary wall biosynthesis in both vessels and fibers [3], [4]. In this network, a group of closely-related NACs, namely secondary wall NACs (SWNs), function as the top master switches capable of activating the entire secondary wall biosynthetic program [5]–[16]. SWNs bind to the 19-bp SNBE (Secondary Wall NAC Binding Element) sequences and directly activate the expression of not only downstream transcription factors but also a number of genes involved in secondary wall biosynthesis and programmed cell death [17]–[19]. Among the SWN-activated transcription factors, MYB46/MYB83 in Arabidopsis and their orthologs in other species act as the second-level master switches that are also able to activate the entire secondary wall biosynthetic program [20]–[28]. Two other MYBs, MYB58 and MYB63 that are direct targets of MYB46/MYB83, have been shown to specifically regulate the expression of genes in the lignin biosynthetic pathway [28]. The available evidence indicates that the transcriptional regulation of secondary wall biosynthesis involves a multi-leveled feed-forward loop regulatory structure, in which SWN and MYB master switches together with their downstream transcription factors function concertedly in the activation of secondary wall biosynthetic genes for cellulose, xylan and lignin [17], [29].

In Arabidopsis, five SWN genes, including VND6 (VASCULAR-RELATED NAC DOMAIN PROTEIN6), VND7, NST1 (NAC SECONDARY WALL THICKENING PROMOTING FACTOR1), NST2 and SND1 (SECONDARY WALL-ASSOCIATED NAC DOMAIN PROTEIN1), have been functionally characterized. VND6 and VND7 are specifically expressed in vessels and their dominant repression causes an inhibition of secondary wall thickening in vessels, indicating that they are transcriptional regulators of secondary wall biosynthesis in vessels [5]. Simultaneous mutations of NST1 and NST2 cause a loss of secondary wall thickening in anther endothecium, leading to an anther dehiscence defect [6]. NST1 and SND1 function redundantly in regulation of secondary wall biosynthesis in both xylary fibers and interfascicular fibers [8], [9]. Overexpression of any of these five SWNs is able to activate secondary wall biosynthetic genes for cellulose, xylan and lignin, leading to ectopic deposition of secondary walls in normally parenchymatous cells. It is evident that Arabidopsis recruited multiple SWNs as transcriptional activators of secondary wall biosynthesis in various secondary wall-forming cell types [3].

Five additional VND genes, namely *VND1*, *VND2*, *VND3*, *VND4* and *VND5*, have previously been shown to be upregulated during xylem differentiation in Arabidopsis *in vitro* cultured cells [5]. Promoter-reporter gene analysis revealed the expression of *VND1*, *VND2* and *VND3* genes in procambial cells and that of *VND4* and *VND5* in vessels. However, no ectopic deposition of secondary walls was observed in the overexpressors of these VNDs [5]. Therefore, it is unclear whether VND1 to VND5 are involved in regulating secondary wall biosynthesis or other processes during xylem development. In this report, we show that *VND1* to *VND5* are specifically expressed in vessels, and when overexpressed, they are able to activate the expression of secondary wall biosynthetic genes for cellulose, xylan and lignin and concomitantly induce ectopic deposition of secondary walls. We also demonstrate that VND1 to VND5 are capable of complementing the secondary wall defects in the *snd1 nst1* double mutant when expressed under the SND1 promoter. Furthermore, transactivation analysis revealed that VND1 to VND5 were able to activate β-glucuronidase (GUS) reporter gene expression driven by SNBE. Our results establish that VND1 to VND5 are SWNs involved in the transcriptional activation of secondary wall biosynthesis in vessels.

## Results

### *VND1* to *VND5* are specifically expressed in developing vessels

**Figure 1:**
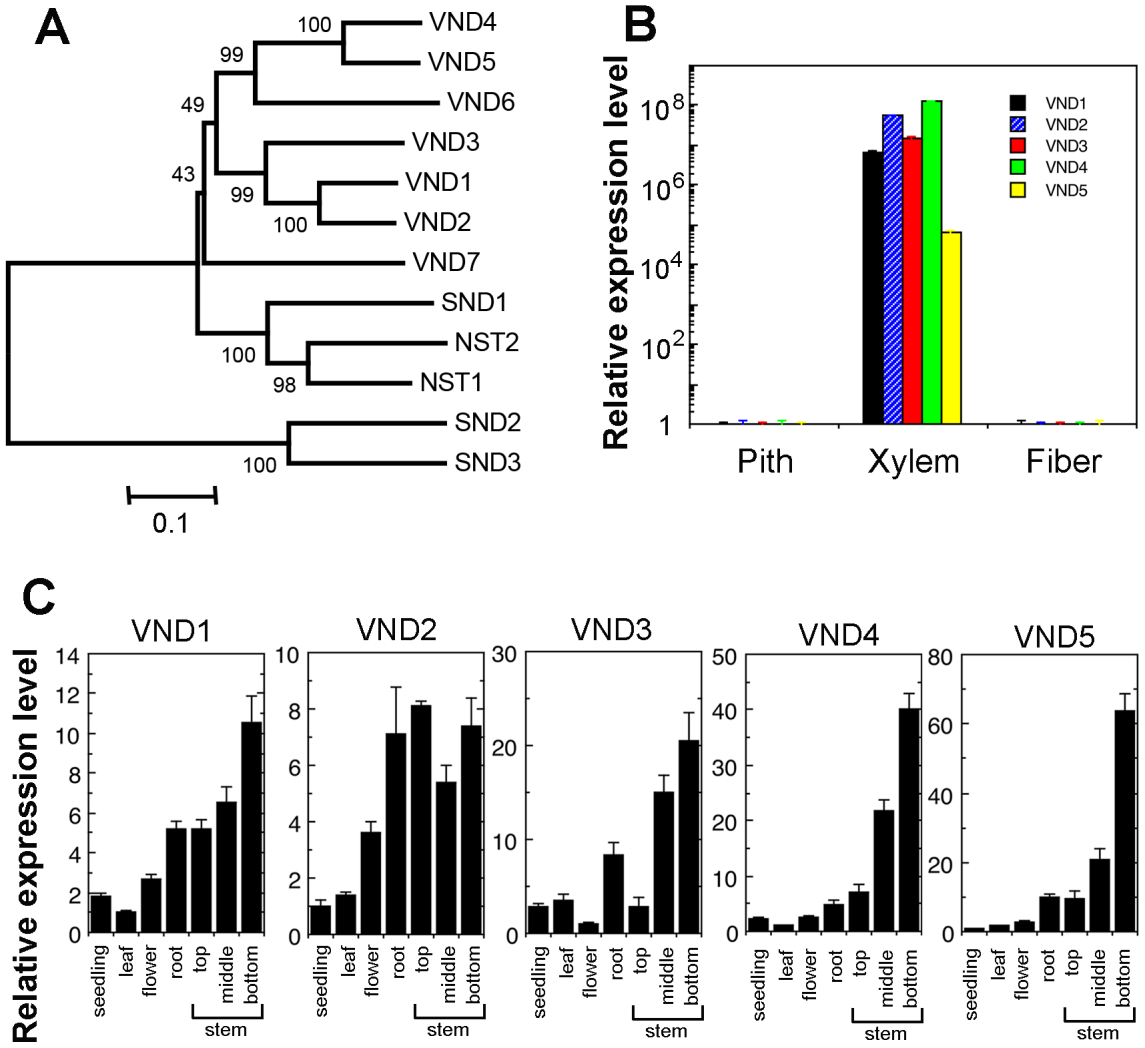
Phylogenetic and gene expression analyses of VNDs. (A) Phylogenetic relationship of VND1 to VND5 together with other Arabidopsis secondary wall NACs. The phylogenetic tree was constructed with the neighbor-joining algorithm using the neighbor-joining method in MEGA5.2 (Tamura et al., 2011). Bootstrap values resulted from 1,000 replicates are shown at the nodes. (B) Quantitative PCR analysis showing the expression of *VND1* to *VND5* in different cell types isolated from Arabidopsis inflorescence stems. The expression of each *VND* in pith cells was set to 1. (C) Quantitative PCR analysis showing the expression of *VND1* to *VND5* in different organs. The expression of each *VND* in the organ with the lowest expression was set to 1. Error bars denote SE of three biological replicates.

**Figure 2:**
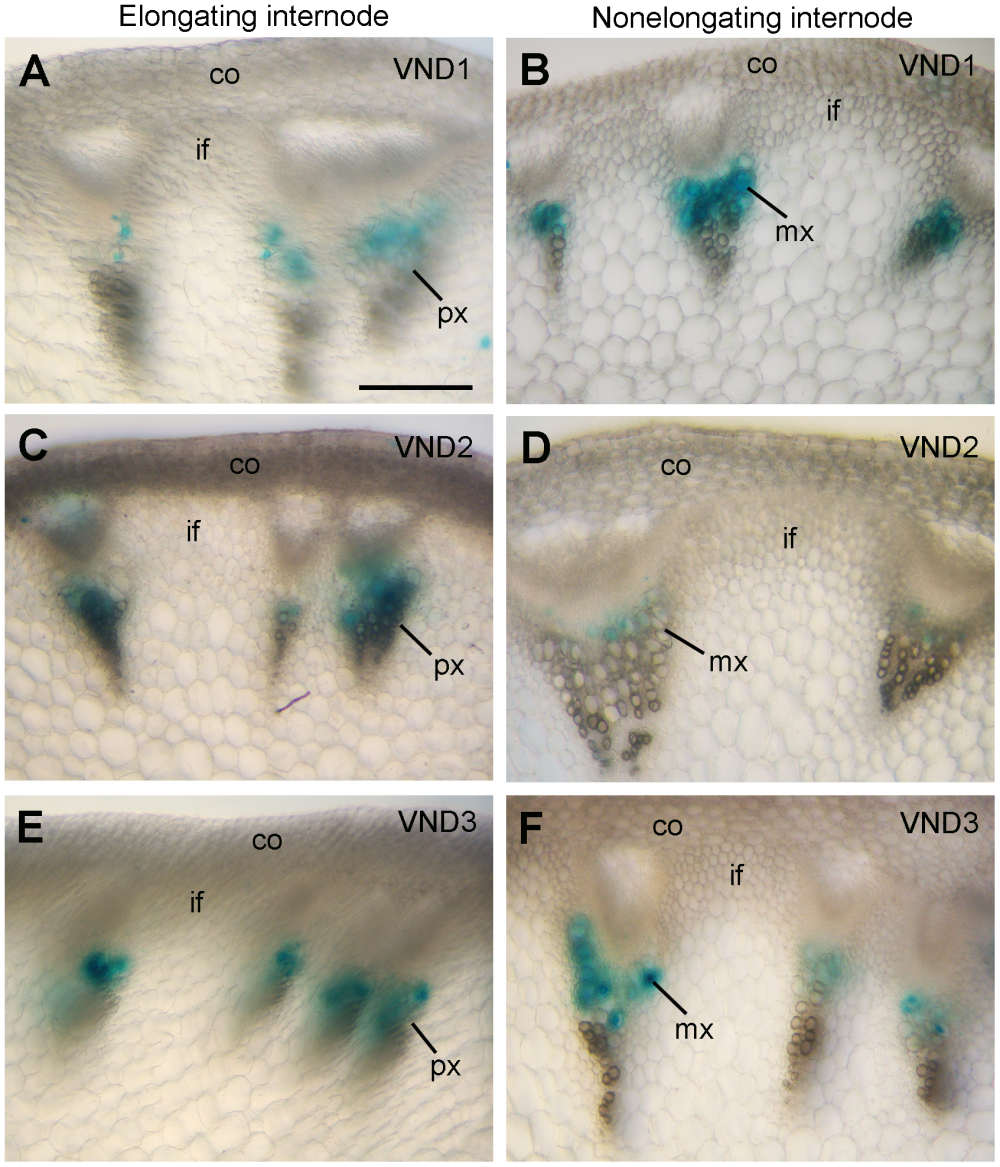
The expression patterns of *VND1*, *VND2* and *VND3* in Arabidopsis stems. The expression constructs containing the *VND1*, *VND2* and *VND3* genes linked with the GUS reporter gene were transformed into the Arabidopsis plants and the first generation of transgenic plants was examined for GUS activity. (A, C, E) Cross sections of elongating internodes showing GUS staining (blue) for *VND1* (A), *VND2* (C) and *VND3* (E) only in developing vessels in the protoxylem. (B, D, F) Cross sections of non-elongating internodes showing GUS staining for *VND1* (B), *VND2* (D) and *VND3* (F) in developing vessels in the metaxylem. co, cortex; if, interfascicular fiber; mx, metaxylem; px, protoxylem. Bar in (A)  = 115 µm for (A) to (F).

**Figure 3:**
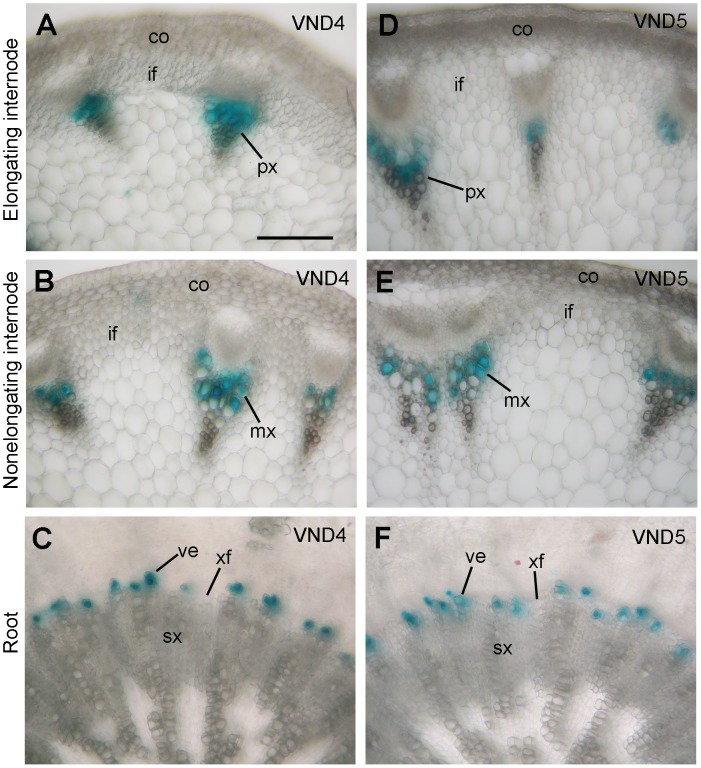
The expression patterns of *VND4* and *VND5* in Arabidopsis stems and the root-hypocotyl region. The expression constructs containing the *VND4* and *VND5* genes linked with the GUS reporter gene were transformed into the Arabidopsis plants and the first generation of transgenic plants was examined for GUS activity. (A, D) Cross sections of elongating internodes showing GUS staining (blue) for *VND4* (A) and *VND5* (D) only in developing vessels in the protoxylem. (B, E) Cross sections of non-elongating internodes showing GUS staining for *VND4* (B) and *VND5* (E) in developing vessels in the metaxylem. (C, F) Cross sections of the root-hypocotyl region showing GUS staining in developing vessels in the secondary xylem. co, cortex; if, interfascicular fiber; mx, metaxylem; px, protoxylem; sx, secondary xylem; ve, vessel; xf, xylary fiber. Bar in (A)  = 96 µm for (A) to (F).

*VND1* to *VND5* genes are closely grouped together with SWNs, including *VND6*, *VND7*, *NST1*, *NST2* and *SND1* (Fig. 1A). Expression analysis showed that they were all expressed in various Arabidopsis organs, among which the stems exhibited the highest level of expression (Fig. 1C). Examination of their expression in laser-microdissected cells from stems revealed that *VND1* to *VND5* were specifically associated with xylem cells but not interfascicular fibers or pith cells (Fig. 1B). To further reveal their expression patterns at the cell-type level, we generated VND-GUS reporter gene constructs and transformed them into wild-type Arabidopsis plants. To ensure that the VND-GUS reporter gene expression represents the endogenous VND expression patterns, a 3-kb 5′ upstream sequence, the entire exon and intron region and a 2-kb 3′ downstream sequence of each *VND* gene was used to create the VND-GUS reporter constructs. Examination of the stems of transgenic plants revealed that the GUS staining for *VND1* to *VND5* was evident only in the developing vessels of protoxylem in elongating internodes (Fig. 2A, C, E and Fig. 3A, B). At this stage of the stem development, secondary wall thickening is only seen in the protoxylem but not in the interfascicular fibers [30]. In nonelongating internodes of stems in which secondary wall thickening occurs in both xylem and interfascicular fibers [30], the GUS staining for *VND1* to *VND5* was prominent in developing vessels in the metaxylem (Fig. 2B, D, F and Fig. 3D, E). In the root-hypocotyl region where extensive secondary growth occurs, the GUS staining for *VND4* and *VND5* was specifically seen in developing vessels but not in xylary fibers in the secondary xylem (Fig. 3C, F). GUS staining for *VND1* to *VND3* was not evident in the root-hypocotyl region. At least 10 independent transgenic lines showing GUS staining for each construct were examined and all of them had vessel-specific expression albeit with different GUS staining intensity. These results demonstrate that the *VND1* to *VND5* genes are specifically expressed in vessels, an expression pattern similar to that of *VND6* and *VND7*. The discrepancy of *VND* expression patterns between this work and a previous report [5] is most likely due to the *VND* sequences used for GUS reporter gene assay. The previous work used only the *VND* promoter sequences [5], which may not contain all the cis-elements required for vessel-specific expression since cis-elements for tissue-specific expression may reside intragenically [31]. We used in this work the whole *VND* gene sequences, which should contain all the cis-elements required for their spatial expression.

### Overexpression of VND1 to VND5 causes ectopic deposition of secondary walls

**Figure 4:**
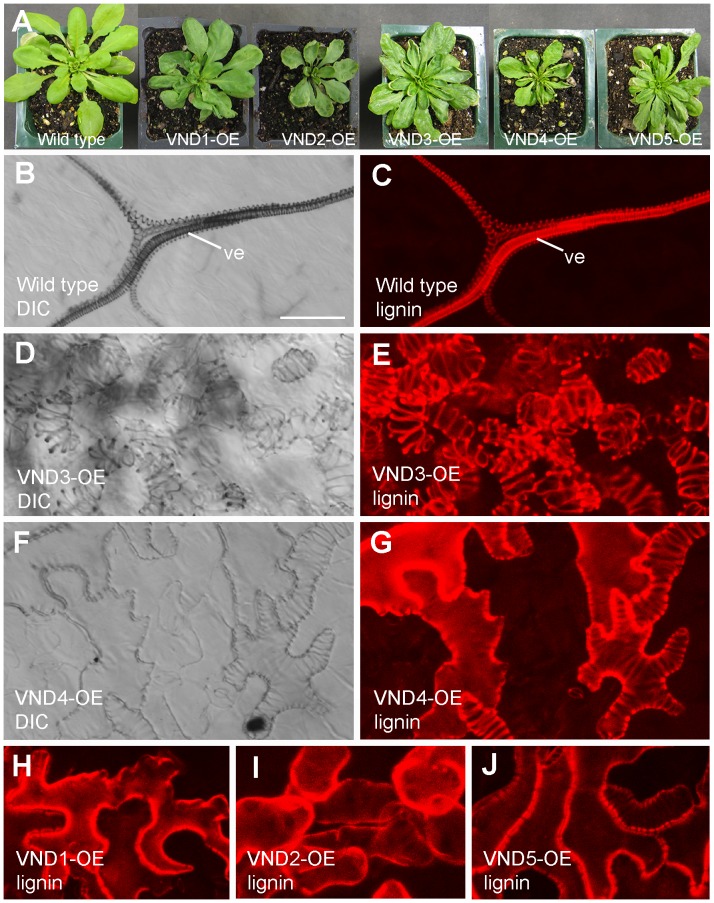
Ectopic deposition of lignified secondary walls in epidermal and mesophyll cells in leaves of VND1 to VND5 overexpressors. (A) Three-week-old plants of the wild type and VND overexpressors (VND-OE). Note the upward curled leaves in the VND overexpressors. (B) and (C) Differential interference contrast (DIC) image (B) and lignin autofluorescence image (C) of a wild-type leaf. Note that lignified secondary walls were only present in veins (ve). (D) and (E) DIC image (D) and lignin autofluorescence image (E) of leaf mesophyll cells of VND3 overexpressors showing ectopic wall thickening and the corresponding lignin signal, respectively. (F) and (G) DIC image (F) and lignin autofluorescence image (G) of the leaf epidermis of VND4 overexpressors showing ectopic wall thickening and the corresponding lignin signal, respectively. (H) to (J) Ectopic lignin deposition in leaf epidermal (H and J) and mesophyll (I) cells in VND1 (H), VND2 (I) and VND5 (J) overexpressors. Bar in (B)  = 29 µm for (B) to (G).

**Figure 5:**
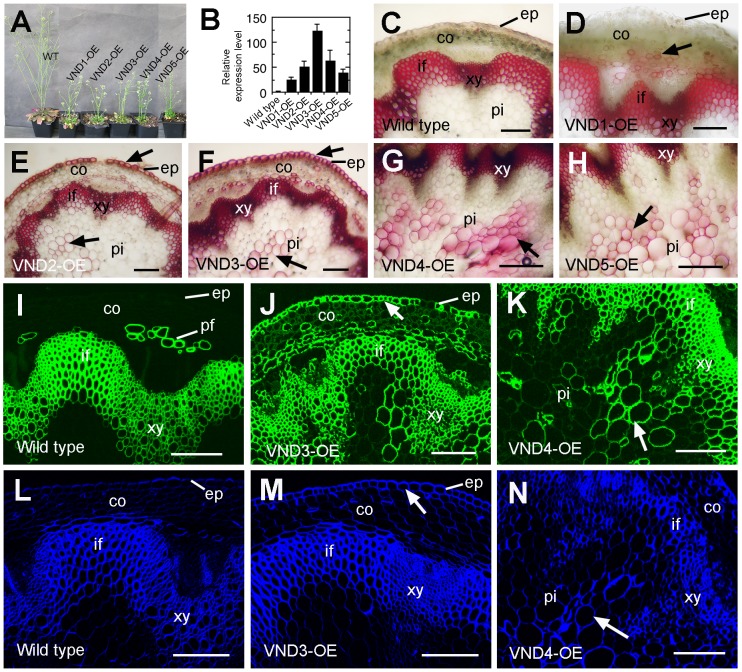
Overexpression of VND1 to VND5 causes ectopic deposition of secondary wall components in transgenic Arabidopsis stems. Cross sections of stems were stained for lignin with phloroglucinol-HCl, xylan with the LM10 xylan antibody, and cellulose with Calcofluor White. (A) VND1 to VND5 overexpression caused a reduction in shoot growth compared with the wild type. (B) Quantitative PCR analysis showing *VND* expression in their overexpression lines. The expression level of corresponding *VND* genes in the wild type is set to 1. Error bars denote SE of three biological replicates. (C) to (H) Lignin staining of stem sections showing ectopic lignin deposition in epidermis, cortical cells or pith cells (arrows) in VND overexpressors (D to H) compared with the wild type (C). (I) to (K) Xylan staining of stem sections showing ectopic xylan deposition in epidermal and cortical cells (arrows) in VND3 (J) and VND4 (K) overexpressors compared with the wild type (I). (L) to (N) Cellulose staining of stem sections showing ectopic cellulose deposition in epidermal and cortical cells (arrows) in VND3 (M) and VND4 (N) overexpressors compared with the wild type (L). co, cortex; ep, epidermis; if, interfascicular fiber; pf, phloem fiber; pi, pith; xy, xylem. Bars  = 220 µm in (C) to (H) and 182 µm in (I) to (N).

To find out whether VND1 to VND5 are transcriptional activators of secondary wall biosynthesis, we investigated whether their overexpression could induce ectopic secondary wall deposition. The full-length cDNAs of *VNDs* were ligated downstream of the cauliflower mosaic virus (CaMV) 35S promoter in a binary vector and the expression constructs were transformed into wild-type Arabidopsis plants. It was noticed that the transgenic seedlings of VND overexpressors had smaller rosettes with curly leaves (Fig. 4A), a phenotype similar to that observed in SND1 overexpressors [7]. This finding indicated that similar to SND1, overexpression of VND1 to VND5 might induce ectopic deposition of secondary walls, thus causing the curly leaf phenotype. To verify this possibility, we examined the curly leaves of VND overexpressors for ectopic secondary walls. In wild-type leaves, secondary wall thickening and lignification were only seen in the veins (Fig. 4B, C). In contrast, ectopic secondary walls with helical and reticulated patterns were evident in many mesophyll and epidermal cells of VND overexpressors (Fig. 4D to J). In addition to the smaller rosettes, VND1 to VND5 overexpressors also had shorter inflorescence stems (Fig. 5A). Cross sections of stems of VND overexpressors showed ectopic deposition of secondary walls in many normally parenchymatous cells compared to the wild type in which secondary wall deposition was only seen in the xylem and interfascicular fibers (Fig. 5B to M). Further histological staining (Fig. 5H to M) revealed that VND overexpression induced ectopic deposition of all the three major secondary wall components, including lignin, xylan and cellulose.

**Figure 6:**
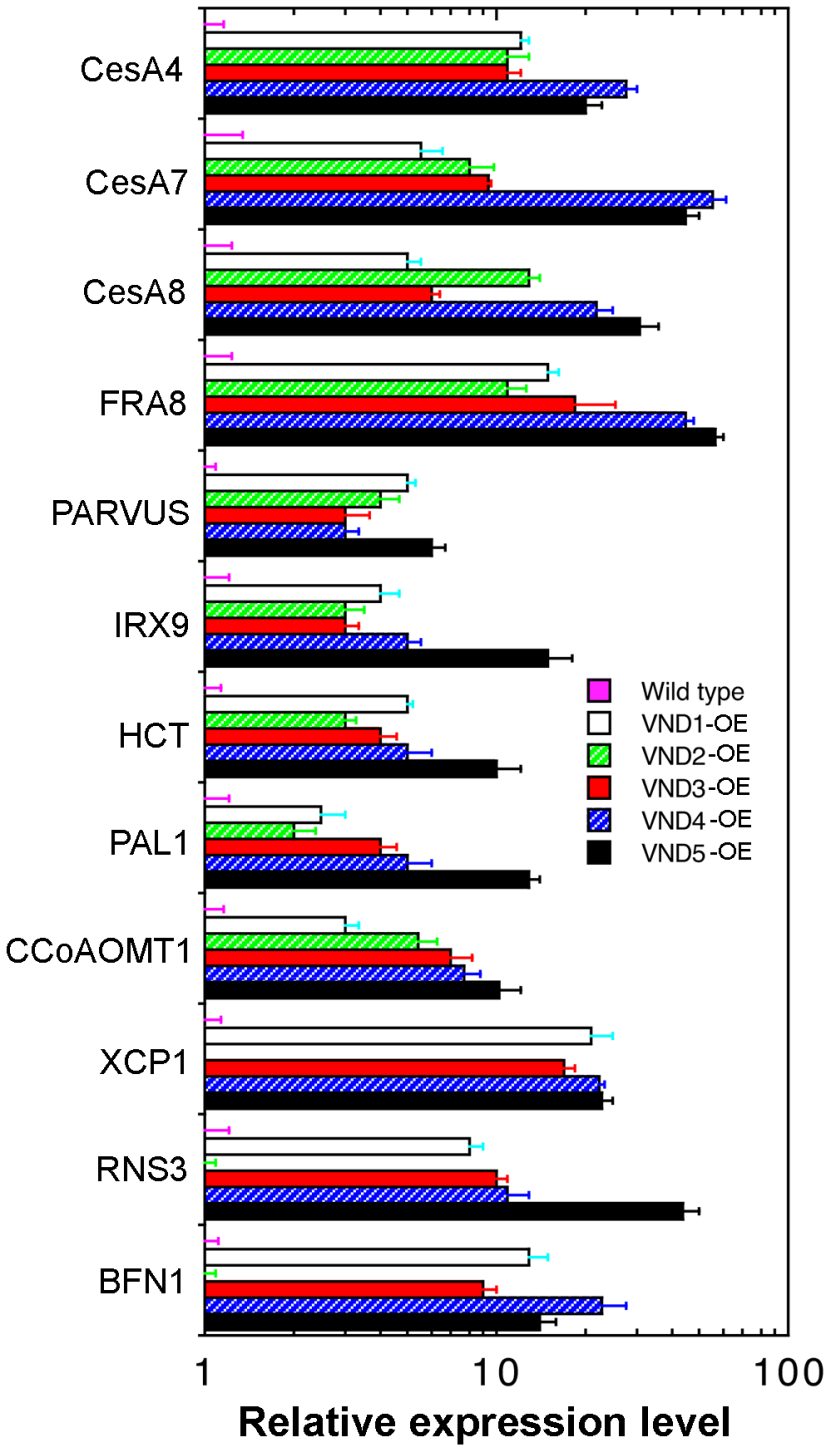
Overexpression of VND1 to VND5 induces the expression of genes involved in secondary wall biosynthesis and programmed cell death. Three-week-old plants of the wild type and VND overexpressors were used for isolation of RNA, which was used for gene expression analysis employing the quantitative PCR approach. The expression level of the gene of interest in the wild type was set to 1. Error bars denote SE of three biological replicates.

Consistent with the ectopic deposition of secondary walls, VND1 to VND5 overexpression resulted in a significant elevation in the expression of secondary wall biosynthetic genes, as evidenced by the induction of nine representative secondary wall genes including three cellulose synthase genes (*CesA4*, *CesA7* and *CesA8*) [32], three genes required for xylan biosynthesis (*FRA8*, *PARVUS* and *IRX9*) [33], [34], [35], and three lignin biosynthetic genes (*CCoAOMT1*, *PAL1* and *HCT*) (Raes et al., 2003, [36]) (Fig. 6). Because ectopic deposition requires induction of all secondary wall biosynthetic genes, it is reasonable to predict that VND overexpression activate all the genes required for ectopic secondary wall deposition. In addition, overexpression of VNDs except VND2 activated the expression of genes involved in programmed cell death as evidenced by the elevation of three representative programmed cell death genes (*XCP1*, *RNS3* and *BFN1*) [37] (Fig. 6), indicating that VNDs also regulate programmed cell death during vessel development.

**Figure 7:**
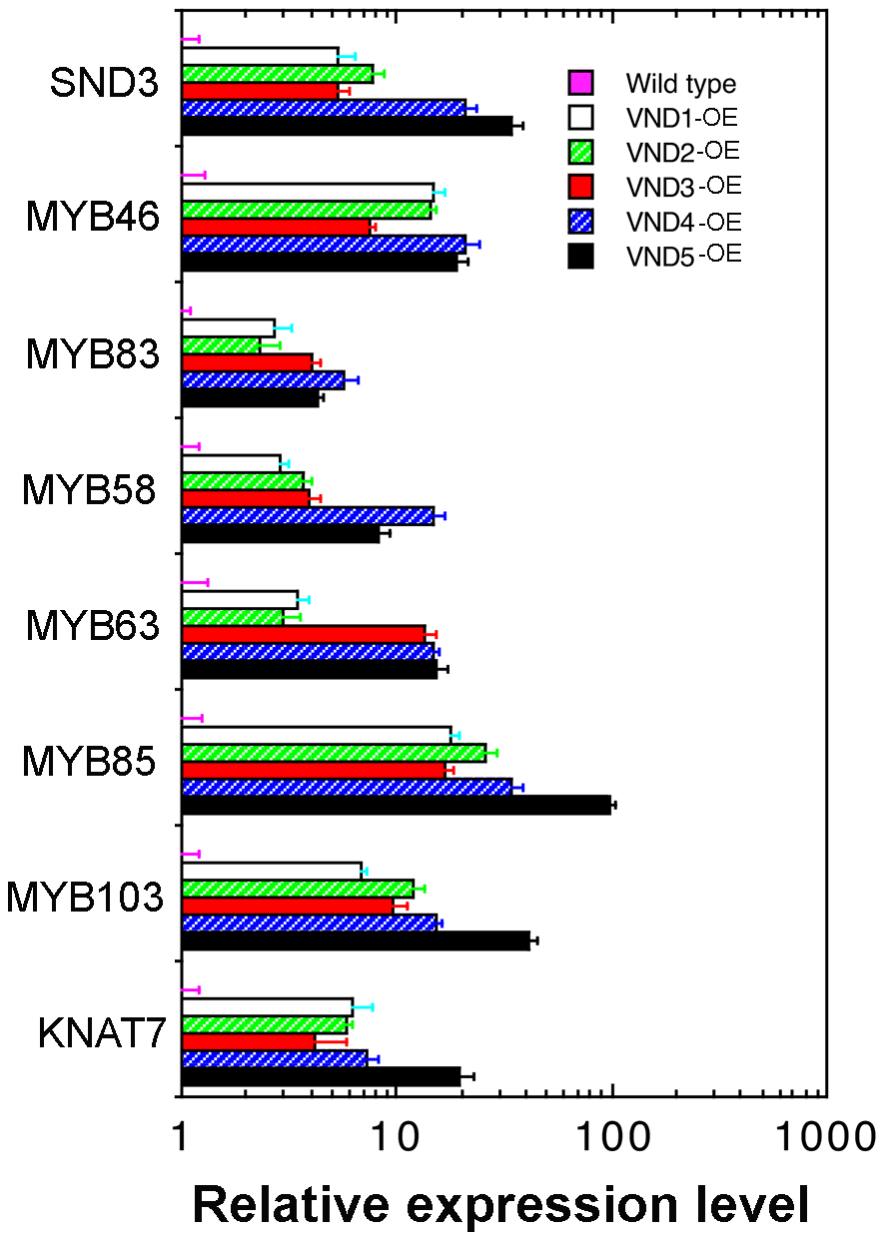
Overexpression of VND1 to VND5 induces the expression of secondary wall-associated transcription factors. Three-week-old plants of the wild type and VND overexpressors were used for isolation of RNA, which was used for gene expression analysis employing the quantitative PCR approach. The expression level of the gene of interest in the wild type was set to 1. Error bars denote SE of three biological replicates.

The finding that VND1 to VND5 are able to induce ectopic deposition of secondary walls prompted us to examine whether they activate the same downstream transcription factors as SND1. A number of transcription factors, including *SND3*, *MYB46*, *MYB83*, *MYB58*, *MYB63*, *MYB85*, *MYB103*, and *KNAT7*, have previously been shown to be induced by SND1 [28], [38]. Quantitative PCR analysis revealed that the expression of these transcription factors was highly induced in VND1 to VND5 overexpressors albeit to various levels (Fig. 7). The increased expression level of corresponding VNDs in their overexpressors was confirmed by quantitative PCR analysis (Fig. 5B). These findings suggest that VND1 to VND5 activate the secondary wall biosynthetic program via the same transcriptional network as SND1.

### Dominant repression of VND3 results in an inhibition of secondary wall thickening in vessels

**Figure 8:**
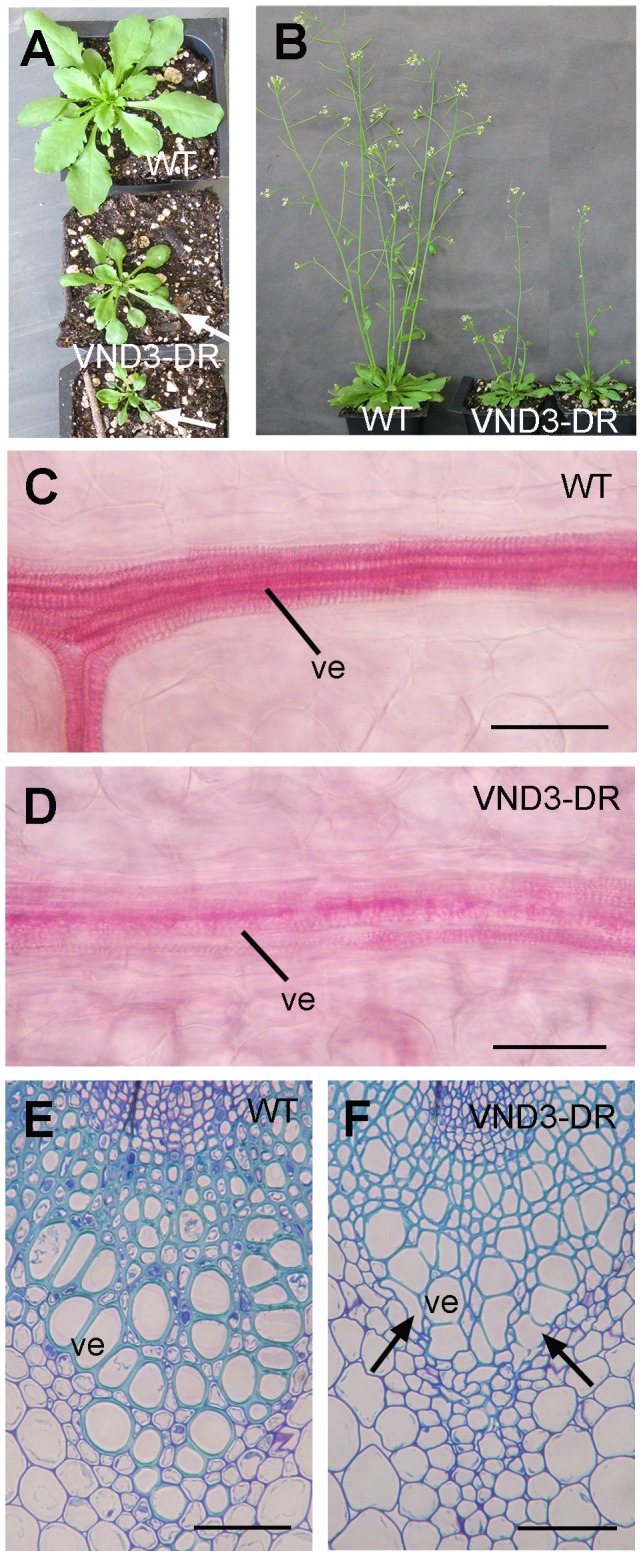
Dominant repression of VND3 causes a reduction in secondary wall thickening and an alteration in vessel morphology in the xylem. (A) Three-week-old plants of the wild type (WT) (top) and VND3 dominant repressors (VND3-DR) (middle and bottom). Note that some leaves of the VND3-DR plants exhibited a wilty phenotype (arrows). (B) Dominant repression of VND3 causes a reduction in shoot growth. (C) and (D) Phloroglucinol staining of leaf veins of the wild type (C) and the VND3 dominant repressor (D). Note the much less-prominent staining of lignified secondary walls in the veins of the VND3 dominant repressor compared with the wild type. (E) and (F) Cross sections of stems showing the xylem bundles of the wild type (E) and the VND3 dominant repressor (F). Note the reduced secondary wall thickness and deformed vessel morphology in the VND3 dominant repressor compared with the wild type. ve, vessel. Bars  = 11 µm in (C) and (D) and 46 µm in (E) and (F).

The results shown above indicate that VND1 to VND5 together with VND6 and VND7 likely play redundant roles in regulating secondary wall biosynthesis in vessels. To further verify the functional roles of VND1 to VND5 in secondary wall biosynthesis, we employed the dominant repression approach to examine the effects of inhibition of VND functions on secondary wall deposition. VND3 was selected as a representative and it was fused with the dominant EAR repressor domain to create the dominant repressor construct, which was transformed into wild-type Arabidopsis. The transgenic plants were confirmed for the expression of VND3 dominant repressor transgene by reverse-transcription PCR. It was found that transgenic plants expressing the VND3 dominant repressor driven by the CaMV 35S promoter had much smaller rosettes and a reduction in shoot growth compared with the wild type (Fig. 8A, B). The rosette leaves also exhibited a mild wilty phenotype, which is reminiscent of that shown by the secondary wall defective mutant, *fra8* [33]. Examination of leaf veins showed that the helical secondary wall thickening in veins was much less prominent in the VND3 dominant repressor than in the wild type (Fig. 8C, D). Furthermore, a deformation of vessels was evident in the stems of the VND3 repressors compared with the wild type (Fig. 8E, F), a phenotype that is common in secondary wall-defective mutants [33], [35], [39].

### VND1 to VND5 are functional homologs of SND1 and NST1

**Figure 9:**
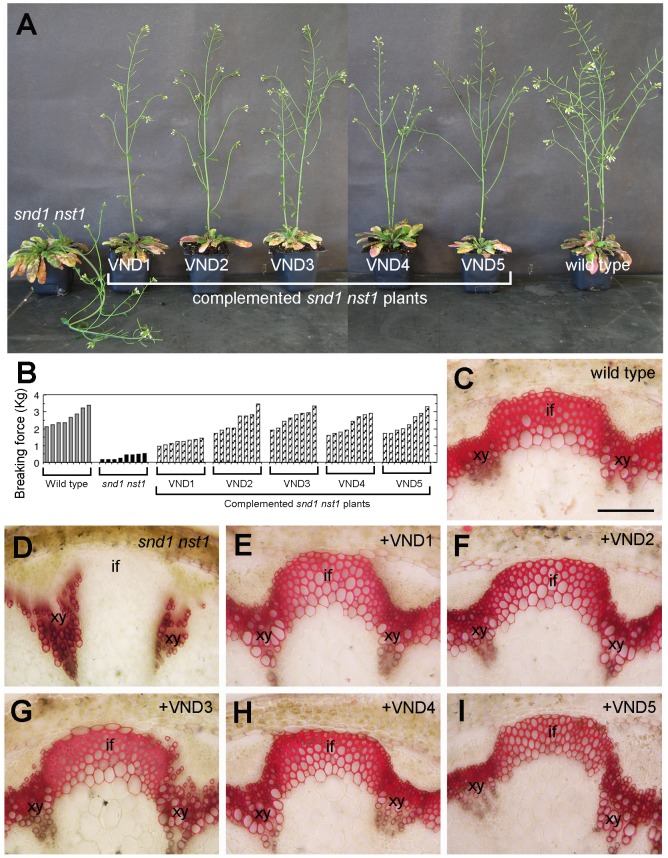
Functional complementation by VND1 to VND5 of the secondary wall defects in the fibers of the Arabidopsis *snd1 nst1* double mutant. (A) Expression of *VND1* to *VND5* driven by the *SND1* promoter rescued the pendent stem phenotype exhibited by *snd1 nst1*. (B) Expression of *VNDs* in *snd1 nst1* resulted in a partial restoration of stem strength. Each bar represents the breaking force of the inflorescence stem of individual plants. (C) Cross section of a wild-type stem showing lignified secondary walls in the xylem and interfascicular fibers. (D) Cross section of an *snd1 nst1* stem showing the lignified secondary walls in the xylem but an absence of lignified secondary walls in the interfascicular fibers. (E) to (I) Expression of VND1 (E), VND2 (F), VND3 (G), VND4 (H) and VND5 (I) in the *snd1 nst1* mutant restored the lignified secondary walls in the interfascicular fibers. The bottom parts of stems of 8-week-old plants were sectioned and stained for lignin with phloroglucinol-HCl. if, interfascicular fiber; xy, xylem. Bar in (C)  = 92 µm in (C) and (I).

The finding that VND1 to VND5 are able to activate the same transcription factors as SND1 suggests that they are functional homologs of SND1. To substantiate this hypothesis, we tested whether expression of VND1 to VND5 could complement the secondary wall defects in the *snd1 nst1* double mutant. Simultaneous mutations of *SND1* and *NST1* genes caused a loss of secondary walls in interfascicular fibers and xylary fibers in stems, leading to a pendent stem phenotype due to reduced stem strength [8], [9]. Expression of VND1 to VND5 driven by the 3-kb SND1 promoter in the *snd1 nst1* double mutant rescued the pendent stem phenotype (Fig. 9A) and partially or fully restored stem strength (Fig. 9B). Cross sections of stems showed that VND1 to VND5 expression in the *snd1 nst1* double mutant restored the formation of lignified secondary walls in fibers (Fig. 9C to I). These results provide another line of evidence demonstrating that VND1 to VND5 are functional homologs of SND1 involved in transcriptional regulation of secondary wall biosynthesis.

### VND1 to VND5 are capable of activating the SNBE-driven GUS reporter gene

**Figure 10:**
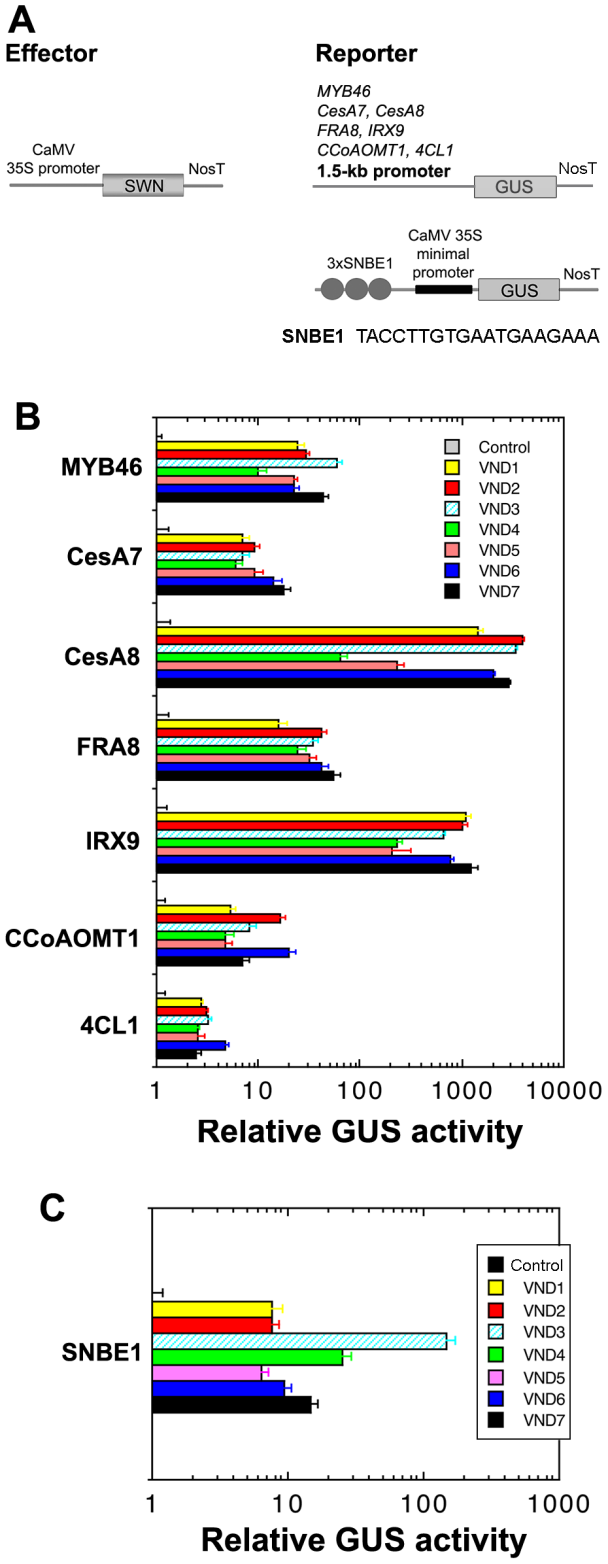
VND1 to VND5 activate the expression of the secondary wall NAC binding element (SNBE)-driven GUS reporter gene. (A) Diagrams of the effector and reporter constructs used for the transactivation analysis. 3xSNBE1, three copies of the 19-bp SNBE1 sequence as shown below the diagram. (B) Transactivation analysis showing the induction by VND1 to VND5 of the GUS reporter gene expression driven by the promoters of *MYB46*, *CesA7, CesA8*, *FRA8, IRX9, CCoAOMT1* and *4CL1*. (C) Transactivation analysis showing that VND1 and VND5 effectively activated the expression of the SNBE-driven GUS reporter gene. Arabidopsis leaf protoplasts were transfected with the reporter and effector constructs and after incubation, the transfected protoplasts were lysed and analyzed for the GUS activity. The control is the GUS activity in protoplasts transfected with the reporter construct and an empty effector construct without VNDs and taken as 1. Error bars are the SE of three biological replicates.

To provide further evidence that VND1 to VND5 activates the expression of genes involved the regulation and biosynthesis of secondary walls, we employed the transactivation analysis in Arabidopsis leaf protoplasts. It was found that VND1 to VND5 significantly induced the expression of the GUS reporter gene driven by the promoter of *MYB46*, a second-level master regulator of secondary wall biosynthesis, and the promoters of six secondary wall biosynthetic genes, *CesA7, CesA8*, *FRA8, IRX9, 4CL1* and *CCoAOMT1* (Fig. 10A, B). These chosen genes for transactivation analysis are induced by VNDs and should represent genes involved in secondary wall biosynthesis. This result corroborates with the finding that VND1 to VND5 are capable of activating the entire program of secondary wall biosynthesis as shown in the VND1 to VND5 overexpressors (Figs. 4–6).

The SWNs, including SND1, NST1, NST2, VND6 and VND7, have previously been demonstrated to bind to a 19-bp semi-palindromic SNBE sequence to activate the expression of their direct target genes [17]. To determine whether VND1 to VND5 also activate their direct targets via the SNBE sites, we tested their ability to activate the SNBE-driven GUS reporter gene. Cotransfection of the VND effector construct and the SNBE-driven GUS reporter construct into Arabidopsis leaf protoplasts revealed that VNDs efficiently activated the SNBE-driven GUS reporter gene (Fig. 10A, C), suggesting that they share similar direct target genes with other SWNs and bind to the SNBE sites to activate their expression.

## Discussion

**Figure 11:**
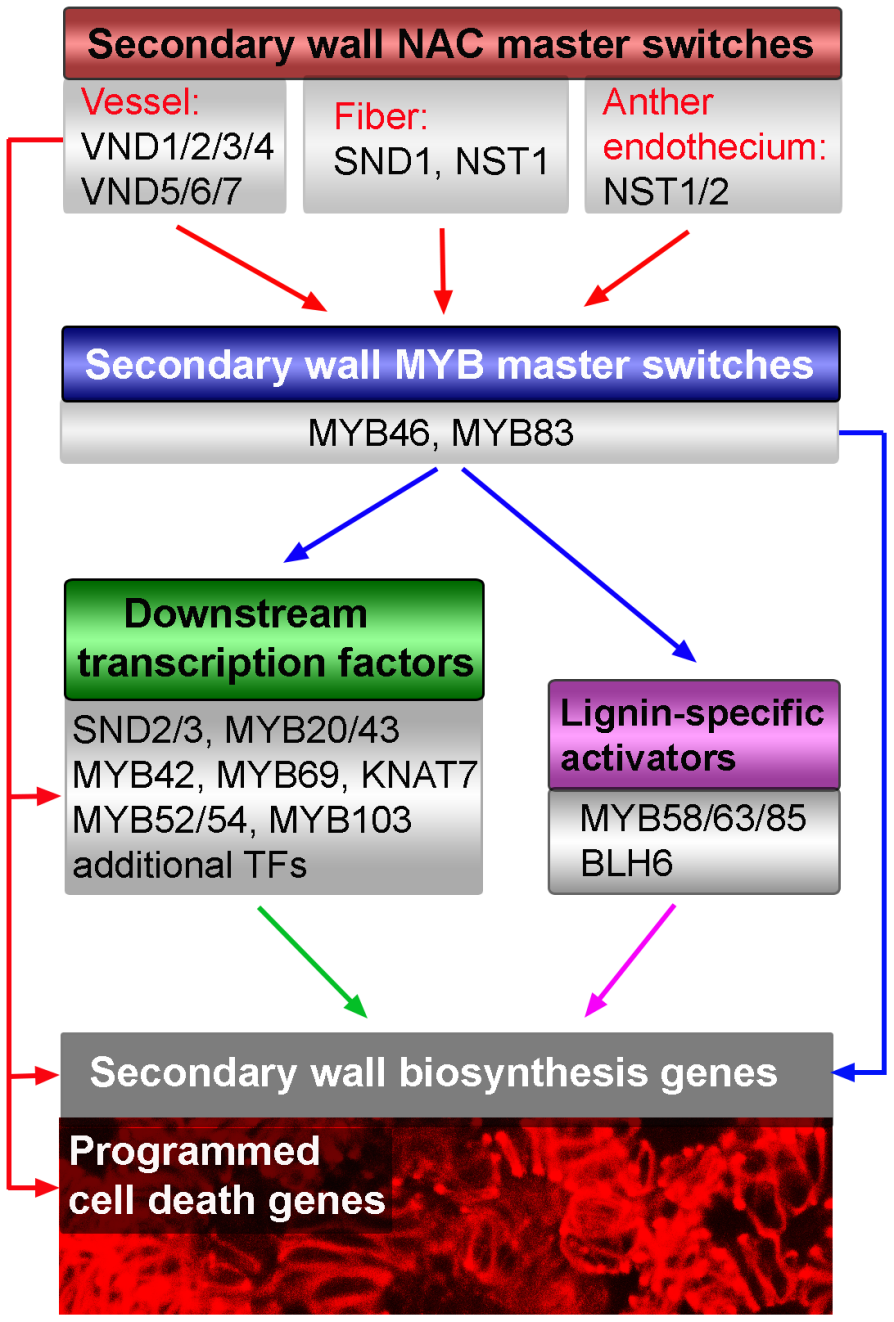
Diagram of the transcriptional network regulating secondary wall biosynthesis in different secondary wall-forming cell types in Arabidopsis. The results from this report add VND1 to VND5 on the list of secondary wall NAC master switches controlling secondary wall biosynthesis. SWNs expressed in various secondary wall-forming cell types activate the second-level MYB master switches and other downstream transcription factors, which together activate secondary wall biosynthetic genes and thereby lead to secondary wall deposition. SWNs also directly activate genes involved in programmed cell death during vessel formation.

Previous studies have demonstrated that five Arabidopsis SWN genes, namely SND1, NST1, NST2, VND6 and VND7, are transcriptional regulators of secondary wall biosynthesis in various secondary wall-forming cell types [3], [4]. The Arabidopsis genome has five additional NAC genes, *VND1* to *VND5*, which belong to the same subgroup as SWNs. Although their expression has been shown to be induced during *in vitro* xylem differentiation [5], the actual functions of these VNDs are unknown. Our present study has established that VND1 to VND5 are vessel-specific secondary wall NACs regulating secondary wall biosynthesis and programmed cell death during vessel development, which further contributes to the complexity of the transcriptional regulation of secondary wall biosynthesis in different cell types (Fig. 11).

We have demonstrated that VND1 to VND5 are capable of activating secondary wall biosynthetic genes and concomitantly inducing ectopic deposition of secondary walls when overexpressed, indicating that they are transcriptional regulators of secondary wall biosynthesis. Our findings provide the first line of genetic evidence that VND1 to VND5 are SWNs controlling secondary wall biosynthesis (Fig. 11). Gene expression analysis showed that similar to that of *VND6* and *VND7* [5], [38], the expression of *VND1* to *VND5* is vessel-specific, indicating that secondary wall formation in vessels is regulated by these 7 functionally redundant VND genes. The high redundancy of VND genes in controlling secondary wall biosynthesis in vessels might signify the importance of vessels in plant survival because secondary wall defects in vessels are detrimental to plant growth [35], [39]. The possibility that VNDs may have differential roles in regulating secondary wall biosynthesis in vessels of different organs and have differential activation strength toward their target genes could not be excluded. Indeed, expression analysis showed that *VNDs* have variations in their expression levels in different organs (Fig. 1C) and in particular, only *VND4* and *VND5* exhibit a high level of expression in vessels of the root-hypocotyl (Fig. 3), indicating that these 7 *VNDs* are expressed at different levels in vessels of different organs. In addition, it has previously been shown that VND6, VND7 and SND1 differentially activate their target genes [17], [18], probably due to their differential binding affinities to the SNBE sites present in the promoters of their target genes. Similarly, VND1 to VND5 may also have differential binding affinities toward SNBE variants and thereby differential activation of their target genes, which may provide a mechanism to fine-tune the cell wall composition of xylem vessels or adapt the developmental program to changing conditions.

We have also demonstrated that VND1 to VND5 are able to restore the secondary wall defects in the *snd1 nst1* double mutant. This finding indicates that although VND1 to VND5 are vessel-specific and SND1 is fiber-specific, they likely activate many of the same downstream target genes and thereby regulate the biosynthesis of secondary walls (Fig. 11). Indeed, expression analysis showed that VND1 to VND5 regulate the expression of the same downstream transcription factors as SND1. Furthermore, they were found to be able to activate the same SNBE sites as SND1. These findings further corroborate the hypothesis that although different SWNs regulate secondary wall biosynthesis in different cell types, they all bind to common SNBE sites albeit with different affinities to differentially activate their direct target genes [17]. This hypothesis is also supported by previous studies showing that SWNs from other species, including poplar, rice, maize, *Brachypodium*, all bind to the SNBE sites [11], [14]–[16].

Secondary wall biosynthesis and programmed cell death are the final two processes during vessel formation. Previous studies of target genes of VND6 and VND7 have shown that both of these processes are regulated by VND6 and VND7 [17]–[19]. Our finding that VND1 to VND5 are also able to induce the expression of genes involved in both secondary wall biosynthesis and programmed cell death further supports the idea that VND1 to VND5 together with VND6 and VND7 function redundantly in regulating secondary wall biosynthesis and programmed cell death during vessel formation. The exception is VND2 that appears to have little activation of programmed cell death genes. Because some SWNs have previously been shown to exhibit differential binding affinities to SNBE variants [17], it is likely that VND2 may have a low binding affinity toward SNBE sites in the promoters of programmed cell death genes and thereby little activation of these genes. Further investigation of how these VND genes are specifically induced during vessel development will enrich our understanding of the molecular mechanisms underlying vascular development.

## Methods

### Quantitative PCR Analysis

Total RNA from different organs and cell types was isolated with a Qiagen RNA isolation kit (Qiagen). The seedlings used were 5 days old. Leaves and roots were from 6-week-old plants. Stems from 6-week-old plants were divided into top, middle and bottom parts, which represent the rapidly elongating internodes, internodes near cessation of elongation and non-elongating internodes, respectively. Different cell types, including interfascicular fibers, xylem and pith cells, were isolated from stems of 6-week-old plants using PALM microlaser system (PALM Microlaser Technologies). First strand cDNAs were synthesized from the total RNA treated with DNase I and then used as a template for PCR analysis. The real-time quantitative PCR was performed with the QuantiTect SYBR Green PCR kit (Clontech) using first strand cDNAs as templates. The PCR primers for *VND1* are 5′-gctcgatgtccataacatcaatgg-3′ and 5′-tcaattatcaaatacgcaaatcccaat-3′, those for *VND2* are 5′-acggattggagagcactcgataaa-3′ and 5′-tcaaacatgtaaatccctatataagtc-3′, those for *VND3* are 5′-accttcaatgcatcgatgatgatc-3′ and 5′-ttagtcttctccactcatcaaaaattg-3′, those for *VND4* are 5′-tggaccaagtcacagactggagag-3′ and 5′-tcacttccatagatcaatctgaca-3′, those for *VND5* are 5′-ttagcaatgatgaagaggctgcag-3′ and 5′-ctagagatcaatctgacaacttga-3′. The PCR primers for secondary wall-associated transcription factors and secondary wall biosynthetic genes were according to Zhong et al. [7]. The relative expression level of each gene was calculated by normalizing the PCR threshold cycle number of each gene with that of the *EF1α* reference gene in each sample. The data were the average of three biological replicates.

### GUS reporter gene analysis

The *VND1* to *VND5* genes containing a 3-kb 5′ upstream sequence, the entire coding region and a 2-kb 3′ downstream sequence were PCR-amplified and used to create the GUS reporter constructs. The GUS gene was inserted in frame right before the stop codon of each *VND* gene. The constructs were transformed into wild-type Arabidopsis by *Agrobacterium*-mediated transformation and the first generation of transgenic plants was analyzed for GUS activity as described previously [33].

### Overexpression and dominant repression

The full-length cDNA of *VND1* (PCR amplified with primers 5′-atggagccaatggaatcttgt-3′ and 5′-tcaattatcaaatacgcaaatcccaat-3′), *VND2* (5′-atggaatcggtggatcaatca-3′ and 5′-tcaaacatgtaaatccctatataagtc-3′), *VND3* (5′-atgatgaaggttgatcaagat-3′ and 5′-ttagtcttctccactcatcaaaaattg-3′), *VND4* (5′-atgaattcattttcccacgtc-3′ and 5′-tcacttccatagatcaatctgaca-3′), and *VND5* (5′-atgaattcgttttcacaagtacct-3′ and 5′-ctagagatcaatctgacaacttga-3′) was ligated downstream of the CaMV 35S promoter in pBI121 to create VND overexpression constructs (VND-OE). The VND dominant repression constructs (VND-DR) were generated by fusing the full-length *VND* cDNA in frame with the dominant EAR repression sequence [40], which was ligated downstream of the CaMV 35S promoter in pBI121. The VND-OE and VND-DR constructs were transformed into wild-type Arabidopsis by *Agrobacterium*-mediated transformation. For each construct, at least 60 transgenic Arabidopsis plants were generated for phenotypic analyses.

### Histology

For light microscopy, stem segments embedded in low viscosity (Spurr's) resin (Electron Microscopy Sciences) were cut into 1-µm-thick sections with a microtome and stained with toluidine blue [41]. Presence of lignin was visualized by staining the sections with phloroglucinol-HCl or using a UV fluorescence microscope [7]. Secondary wall cellulose was stained by incubating sections with 0.01% Calcofluor White [42]. Under the conditions used, only secondary walls exhibited brilliant fluorescence. Xylan was detected by using the monoclonal LM10 antibody against xylan and fluorescein isothiocyanate-conjugated goat anti-rat secondary antibodies [43].

### Complementation of the Arabidopsis *snd1 nst1* mutant

The full-length cDNA of *VND1* to *VND5* driven by the 3-kb *SND1* promoter were cloned into the pGPTV vector and introduced into the Arabidopsis *snd1 nst1* double mutant [8]. More than 60 transgenic plants were generated for each construct and at least 8 complemented *snd1 nst1* double mutant plants that exhibited normal growth as the wild type were chosen for examination of stem strength and restoration of secondary walls in fibers. For stem strength analysis, basal parts of the main inflorescence of 8-week-old plants were measured for the breaking force using a digital force/length tester [44]. The breaking force was calculated as the force needed to break apart a stem segment.

### Transactivation Analysis

To test the ability of VND1 to VND5 to activate the promoters of secondary wall-associated transcription factors and secondary wall biosynthetic genes, the reporter construct containing the GUS reporter gene driven by a 1.5-kb promoter of the gene of interest and the effector construct containing VNDs driven by the CaMV 35S promoter were co-transfected into Arabidopsis leaf protoplasts [45]. To test the ability of VNDs to activate the SNBE site, the reporter construct containing the GUS reporter gene driven by three copies of SNBE sequence and the effector construct containing VNDs driven by the CaMV 35S promoter were co-transfected into Arabidopsis leaf protoplasts. Another construct containing the firefly luciferase gene driven by the CaMV 35S promoter was included in each transfection for determination of the transfection efficiency. After 20-hr incubation, protoplasts were lysed and the supernatants were subjected to assay of the GUS and luciferase activities [46]. The GUS activity was normalized against the luciferase activity in each transfection, and the data are the average of three biological replicates.

### Statistical Analysis

The experimental data of the quantitative PCR analysis and GUS activity assay were subjected to statistical analysis using the Student's *t* test program (http://www.graphpad.com/quickcalcs/ttest1.cfm), and the quantitative difference between the two groups of data for comparison in each experiment was found to be statistically significant (p<0.001).

### Accession numbers

The Arabidopsis Genome Initiative locus identifiers for genes used in this study are VND1 (At2g18060), VND2 (At4g36160), VND3 (At5g66300), VND4 (At1g12260), VND5 (At1g62700), SND1 (At1g32770), NST1 (At2g46770), NST2 (At3g61910, VND6 (At5g62380), VND7 (At1g71930), SND2 (At4g28500), and SND3 (At1g28470).
